# Neurons in primary visual cortex represent distribution of luminance

**DOI:** 10.14814/phy2.12966

**Published:** 2016-09-21

**Authors:** Yong Wang, Yi Wang

**Affiliations:** ^1^ State Key Laboratory of Brain and Cognitive ScienceInstitute of BiophysicsChinese Academy of SciencesBeijingChina; ^2^ University of Chinese Academy of SciencesBeijingChina

**Keywords:** Extracellular recording, luminance distribution, neural coding, primary visual cortex

## Abstract

To efficiently detect a wide range of light‐intensity changes, visual neurons must adapt to ambient luminance. However, how neurons in the primary visual cortex (V1) code the distribution of luminance remains unknown. We designed stimuli that represent rapid changes in luminance under different luminance distributions and investigated V1 neuron responses to these novel stimuli. We demonstrate that V1 neurons represent luminance changes by dynamically adjusting their responses when the luminance distribution changes. Many cells (35%) detected luminance changes by responding to dark stimuli when the distribution was dominated by bright stimuli, bright stimuli when dominated by dark stimuli, and both dark and bright stimuli when dominated by intermediate luminance stimuli; 13% of cells signaled the mean luminance that was varied with different distributions; the remaining 52% of cells gradually shifted the responses that were most sensitive to luminance changes when the luminance distribution varied. The remarkable response changes of the former two cell groups suggest their crucial roles in detecting luminance changes. These response characteristics demonstrate that V1 neurons are not only sensitive to luminance change, but also luminance distribution change. They encode luminance changes according to the luminance distribution. Mean cells represent the prevailing luminance and reversal cells represent the salient stimuli in the environment.

## Introduction

Light varies over a wide range of intensity by a factor of at least 10^9^ during the 24‐h day/night cycle (Rieke and Rudd 2009; Wark et al. 2009). To cope with this enormous variation in ambient luminance, responses of visual neurons are highly adaptive, increasing their responses when the luminance signal is weak to maintain response sensitivity and decreasing their responses when the luminance signal is strong to prevent response saturation (Baccus and Meister 2004; Rieke and Rudd 2009; Wark et al. 2007, 2009; Carandini and Heeger 2012). Light adaptation largely occurs in the photoreceptor cells of the retina (Sakmann and Creutzfeldt 1969; Boynton and Whitten 1970; Normann and Perlman 1979; Schneeweis and Schnapf 1999; Rieke and Rudd 2009), while contrast and orientation adaptations mainly occur in the cells of the retina, the lateral geniculate nucleus (LGN) in the thalamus, and the visual cortex (Ohzawa et al. 1982; Bonds 1991; Muller et al. 1999; Brown and Masland 2001; Baccus and Meister 2004; Solomon et al. 2004; Kohn 2007; Rieke and Rudd 2009; Wark et al. 2009; Li et al. 2011; Carandini and Heeger 2012). V1 neurons are sensitive to luminance changes (Bartlett and Doty 1974; Rossi et al. 1996; Kinoshita and Komatsu 2001; Peng and van Essen 2005; Geisler et al. 2007; Hung et al. 2007; Dai and Wang 2012; Li and Wang 2013). However, it is largely unknown whether and how these V1 neurons adapt their responses when the distribution of luminance varies. Therefore, investigating V1 neuron responses to the change of luminance distribution is important to our understanding of the mechanisms underlying our visual perception when faced with large changes in ambient luminance.

The visual world is full of textured stimuli. V1 neurons respond to luminance changes presented by stimuli such as gratings (Geisler et al. 2007; Li and Wang 2013). Because many V1 neurons do not respond to uniform luminance stimuli (Dai and Wang 2012), we used grating stimuli to effectively activate the neurons in the current study. During natural vision, eye movements often lead to rapid changes of luminance in the receptive field (RF) of a neuron. Moreover, the ambient luminance can also change drastically in many situations, for instance, when one moves from outdoor sunshine to indoor dim light. Therefore, the local luminance that individual V1 neurons are exposed to is continuously changing, and the global distribution of luminance distribution that is experienced by these neurons also varies. We investigated the response properties of V1 neurons to luminance variations under different luminance distributions and found that the neurons coded luminance distribution in the environment.

## Materials and Methods

### Animal preparation

All experiments were conducted in accordance with the guidelines of NIH and EACUMC (Experimental Animal Care and Usage Management Committee of Beijing City). The protocols were approved by the Institutional Animal Care and Usage Committee of the Institute of Biophysics, Chinese Academy of Sciences. We confirmed that the procedures for animal preparation complied with the ethics policy of the *Journal of Physiology* (*J. Physiol*. 2015, 593(12):2547–2549). Seventeen adult cats (2–3 kg) were used. The animals were ordered from XingLong Experimental Animal Breeding Plant at HaiDian in Beijing. An animal was prepared for single‐unit recording as described previously (Dai and Wang 2012). The animal was initially sedated with ketamine (20–30 mg kg^−1^, i.m.), followed by an injection of dexamethasone and atropine (i.m.). Then, the trachea and forelimb vein were cannulated. The animal was anesthetized with a rapid infusion of sufentanil (1.2 *μ*g kg^−1^, i.v.) and propofol (1.2 mg kg^−1^, i.v.) and was artificially respired. Surgery was performed under deep intravenous anesthesia. During recording, anesthesia was maintained by continuously infusing sufentanil (0.15–0.22 *μ*g kg^−1^ h^−1^, i.v.) and propofol (1.8–2.2 mg kg^−1 ^h^−1^, i.v.) combined with gallamine triethiodide (for paralysis, 10 mg kg^−1^ h^−1^, i.v.) in a physiological solution containing 5% glucose. The anesthetic depth of the animal was monitored by end‐tidal CO_2_, ECG, and EEG. The anesthetic depth was also judged by pinching its toes or ears and monitoring changes in its heart rate and/or muscle tone. The infusion rate was accordingly adjusted to maintain an appropriate anesthetic level. Body temperature was maintained at 38°C by a heating pad. Homatropine was applied to dilate the pupils, and phenylephrine hydrochloride was used to retract the nictitating membranes. Following data collection, the animals were deeply anesthetized and sacrificed via an overdose of pentobarbital sodium (60 mg kg^−1^, i.v.).

### Extracellular recording

Rigid gas‐permeable contact lenses with a power of +2.0 D and 3 mm artificial pupils covered the corneas to prevent desiccation and to focus the eyes on a cathode ray tube (CRT) monitor that was 57 cm away. A 2.5 mm × 2.5 mm craniotomy was centered at Horsley‐Clarke P 2.5 mm and L 2.5 mm (Tusa et al. 1978). One of the eyes was covered. A glass‐coated tungsten microelectrode (1–3 MΩ) was driven by a microdrive (Narishige [Narishige, Tokyo, Japan]) into V1 (area 17). Extracellular potentials that were driven by stimulation of the RFs of V1 cells through the dominant eye were recorded from the cortical region that represented the central visual field. Recorded neurons were discriminated based on RF properties, which included RF position in the visual field (the lower quadrant of the contralateral central visual field with an eccentricity of approximately 5–10°), RF size (most of them were 1–4°), and the preferred spatial frequencies that were higher relative to cells in V2 (area 18) (Nishimoto et al. 2005). Usually, when a cell was tested, the preliminary testing for its RF parameters and responses to luminance changes in the subsequent quantitative experiments lasted for approximately 2–4 h. Unit activities of V1 cells were acquired with a TDT amplifier and OpenEX software (Tucker‐Davis Technologies Inc, Alachua, FL, USA) and were sampled at 12 kHz. Single units were identified post hoc and offline with a TDT OpenSorter (Tucker‐Davis Technologies Inc).

### Visual stimulation

Stimuli were presented by an Iiyama CRT monitor (HM204DT A, 800 × 600 pixels, 40°×30° in visual angle) (iiYAMA Corporation, Tokyo, Japan) with a refresh rate of 100 Hz. The monitor was calibrated by the gamma correction to remove luminance nonlinearities. To control illumination, the space between the monitor and the animal and the space around the animal were covered by black boards. The luminance of the experiment room was 0.1 cd m^−2^ (measured by a ColorCAL colorimeter [CRS Ltd, Kent, UK]). The preferred orientation (0°–165° in 15° step) and spatial frequency (SF, 0.1–2.3 cycles per degree) of the cell were measured by presenting static 100% Michelson contrast sinusoidal gratings and its classical RF was measured by presenting white and dark short bars (1.5°×0.5°) with the reverse correlation method (Jones and Palmer 1987; DeAngelis et al. 1993; Ringach et al. 1997; Mazer et al. 2002; Nishimoto et al. 2005; Hu et al. 2011; Li and Wang 2013). The firing rates of the cell to sinusoidal gratings with the preferred SF and orientation that drifted along the preferred direction were measured to calculate the F_1_/F_0_ modulation index. The contrast of the grating stimulus was 100%. In these preliminary tests for the RF properties of a cell, the stimuli were presented on a uniform background with a luminance of 16.7 cd m^−2^, which equaled the mean luminance of all stimuli in a set.

**Figure 1:**
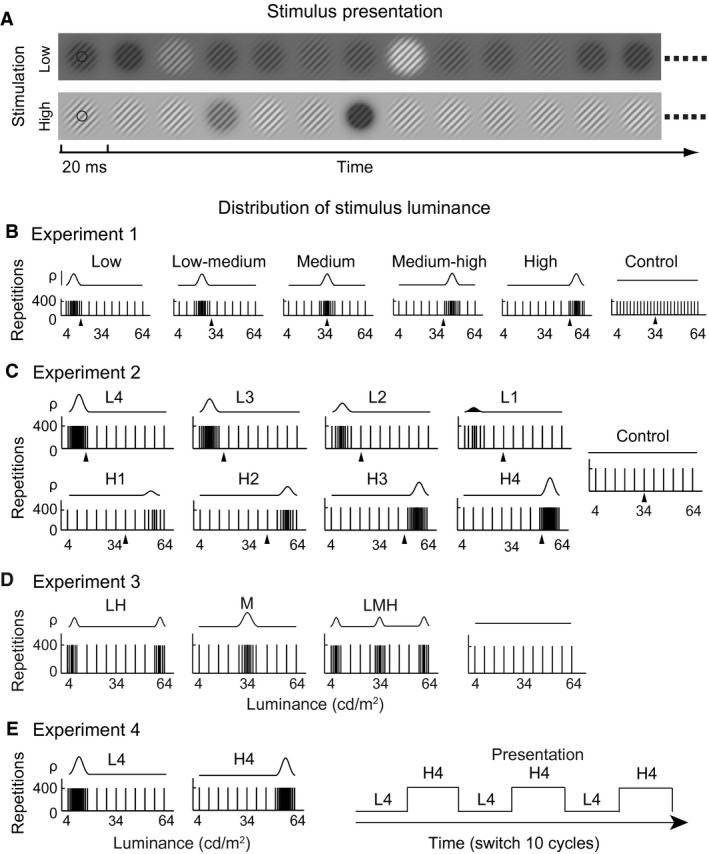
Visual stimulation. (A) Stimulus presentation. Examples of random presentations of Low and High sets of stimuli (B) without interstimulus intervals, respectively, in two separate blocks. Each stimulus was flashed for 20 msec on the background luminance that equaled to the mean luminance of all grating stimuli in a set. The circles represent the stimulated receptive field of a cell. (B–E) Distribution of stimulus luminance in Experiments 1–4. Top row: probability (*ρ*) of a luminance distribution (curve) generated by stimuli (vertical short bars) along the luminance dimension (4–64 cd m^−2^; *x*‐axis). Each bar represents 400 presentations (*y*‐axis). A high density of bars indicates a high percentage of stimuli distributed in the local luminance range. Arrowheads: mean luminance of a stimulus set. In Experiment 1 (B), six sets of stimuli were used. From left to right panel, dense stimuli (83.6%) distributed in 4–16 cd m^−2^ (Low), 16–28 cd m^−2^ (low–medium), 28–40 cd m^−2^ (medium), 40–52 cd m^−2^ (medium–high), and 52–64 cd m^−2^ (high) of the luminance range, and uniform distribution (Control; 4–64 cd m^−2^). In Experiment 2 (C), nine sets of stimuli were applied. The stimulus number increased gradually from 3 of the uniform (control) distribution to 7 (L1), 13 (L2), 23 (L3), and 41 (L4) of a high density of stimuli (HDS) in 4–16 cd m^−2^ of the low luminance range (upper row) and to 7 (H1), 13 (H2), 23 (H3), and 41 (H4) of HDS in 52–64 cd m^−2^ of the high luminance range (bottom row). In Experiment 3 (D), four sets of stimuli with a symmetrical distribution were presented. LH (low–high): 21 stimuli of HDS distributed symmetrically in 4–10 cd m^−2^ and the other 21 in 58–64 cd m^−2^; M (medium): 41 stimuli of HDS distributed symmetrically in 28–40 cd m^−2^; LMH (low–medium–high): 21 stimuli of HDS distributed symmetrically in 4–10 cd m^−2^, the other 21 in 31–37 cd m^−2^, and the remaining 21 in 58–64 cd m^−2^; Control: uniform distribution of 11 stimulus luminances in 4–64 cd m^−2^. In Experiment 4 (E), L4 and H4 sets of stimuli in (C) were alternately presented in the manner indicated by the square wave form at the right panel. Each horizontal line of the wave form was 39.2 sec, during which L4 or H4 was presented for 40 repetitions, and the level of the horizontal lines indicates that HDS was distributed in low (L4) or high (H4) luminance ranges. The L4 and H4 were switched 10 cycles over time.

Responses of V1 cells to the luminance changes of grating stimuli were investigated. To avoid the effects of the contrast at the border of a stimulus in a RF on the cell responses, the stimulus size was five times larger than the RF in diameter, and the stimulus border was blurred by a smooth change in luminance from the stimulus level to the background (Fig. 1A). The grating was defined by the formula:

(1) $$\begin{array}{cc} I(x,y)= & \;\;L+L\cdot C\cdot \;\text{cos}(2\pi \cdot \text{SF}\cdot x\cdot \;\text{cos}(\theta -90)\\ & +2\pi \cdot \text{SF}\cdot y\cdot \text{sin}(\theta -90)-\phi ) \end{array}$$

where the *I*(*x*,*y*) denotes the light intensity at position (*x*,*y*), the *L* is the mean luminance, the *C* is the contrast (Michelson contrast), the SF is the preferred spatial frequency (cycles per degree), the *θ* is the preferred orientation (degree), and the *φ* is the spatial phase (0, *π*/2, *π*, 3 *π*/2) of the gratings.

For each cell, all grating stimuli had its preferred orientation and SF and had the same 70% contrast. The mean luminance of a grating stimulus in a given stimulus set ranged from 4 to 64 cd m^−2^ in all four experiments described below (Fig. 1B–E). A critical parameter in these experiments was that a high density of stimuli (HDS) was concentrated in a narrow range of luminance, and the remaining small number of stimuli was uniformly distributed in the remaining ranges of luminance. In the more central position of the HDS range, the more stimuli that were distributed along the luminance axis, that is, the closer to the center of HDS, the smaller was the luminance difference between two stimuli along the luminance dimension (e.g., Fig. 1B). Different stimulus sets had an HDS located within different ranges of luminance (Fig. 1B). A set of gratings were presented in a pseudorandom sequence without an interstimulus interval, and each stimulus was statically flashed for 20 msec and for 400 repetitions on a uniform background that had a luminance that was always equal to the mean luminance of all gratings in the set of stimuli (Fig. 1A). Different sets of stimuli had different mean luminance and were presented in separate blocks, and the sequence of blocks was random, except for Experiment 4 (Fig. 1E). Four spatial phases (0, *π*/2, *π*, 3 *π*/2) of each sinusoidal grating were included in each stimulus set.

#### Experiment 1

To assess V1 neuron responses to different luminance distributions in which the majority of stimuli were distributed in a narrow range of luminance and the minority of stimuli were distributed in a wide range, five stimulus sets and a control set of stimuli were used (Fig. 1B). Each of the five stimulus sets contained 49 stimuli, in which 83.6% (41/49) of stimuli were within HDS range of luminance (with an SD of 2.89 cd m^−2^), while the other 16.4% (8/49) were uniformly distributed across the remaining luminance range. For instance, in the low distribution stimulus set (left most of Fig. 1B), 83.6% stimuli were in the range of 4–16 cd m^−2^, and 16.4% were in the remaining 16–64 cd m^−2^ range. The other four stimulus sets used the same HDS distribution but differed from each other by the location of HDS in the 16–28 cd m^−2^ (low–medium), 28–40 cd m^−2^ (medium), 40–52 cd m^−2^ (medium–high), or 52–64 cd m^−2^ (high) ranges of luminance (Fig. 1B). The remaining 16.4% of the stimuli were distributed uniformly in the 4–16 and 28–64 cd m^−2^, 4–28 and 40–64 cd m^−2^, 4–40 and 52–64 cd m^−2^, and 4–52 cd m^−2^ ranges, respectively. The control set of stimuli was the 49 stimuli distributed uniformly in 4–64 cd m^−2^. The six sets of stimuli were presented in separate blocks.

#### Experiment 2

To determine whether HDS was a critical factor that caused the response variations of neurons to different luminance distributions, eight stimulus sets in which HDS had different luminance intensities and a control set of stimuli were implemented (Fig. 1C). The control set contained 11 luminance stimuli that were uniformly distributed in 4–64 cd m^−2^. For four sets of stimuli, the HDS was in the low range (4–16 cd m^−2^) of luminance, and the stimulus numbers of HDS stimuli were 7 (L1), 13 (L2), 23 (L3), and 41 (L4 of Fig. 1C). For the other four sets, the HDS was in the high range (52–64 cd m^−2^) of luminance and the numbers of HDS stimuli were 7 (H1), 13 (H2), 23 (H3), and 41 (H4 of Fig. 1C). The nine sets of stimuli were presented in separate blocks.

#### Experiment 3

To determine whether the symmetry of luminance distribution played a role in the response variations of neurons to different luminance distributions, three stimulus sets and a control set of stimuli with a symmetrical distribution of luminance were investigated (Fig. 1D). The first set (LH of Fig. 1D) had 21 stimuli of HDS in the low range (4–10 cd m^−2^) and the other 21 stimuli of HDS in the high range (58–64 cd m^−2^) of luminance (most left panel of Fig. 1D), and 7 stimuli were uniformly distributed in 10–58 cd m^−2^. The second set (M of Fig. 1D) had 41 stimuli of HDS in the medium range (28–40 cd m^−2^) of luminance, and the other 8 stimuli were uniformly distributed in 4–28 and 40–64 cd m^−2^. The third set (low–medium–high of Fig. 1D) had 21 stimuli of HDS in the low range (4–10 cd m^−2^), 21 stimuli of HDS in the medium range (31–37 cd m^−2^), 21 stimuli of HDS in the high range (58–64 cd m^−2^) of luminance (third panel of Fig. 1D), and 6 stimuli were uniformly distributed in the 10–31 and 37–58 cd m^−2^ ranges. The control set was the same as in Experiment 2. The four sets of stimuli were presented in separate blocks.

#### Experiment 4

To exclude the possibility that neuron response variations to different luminance distributions were due to the fluctuation of neuronal responses in separate temporal test blocks, two stimulus sets of the L4 and H4 distributions in Experiment 2 were alternately presented in a block (Fig. 1E). The two sets of stimuli were presented together, in one block, by switching from one to the other after one of them was presented for 40 repetitions (right panel of Fig. 1E). This generated the change of luminance distribution between the low‐HDS and high‐HDS ranges. This switching presentation was recycled 10 times to reach a total 400 repetitions for each set of stimuli.

### Data analyses

We recorded 158 cells in V1 across all cortical layers. Most cells were tested in Experiment 1, and a subgroup of these cells that were maintained to isolate for a sufficient time were further tested in Experiments 2, 3, or 4. The cells that did not last to the end of one recording session and exhibited responses that did not meet the criterion for the significant responses (subsequently described) were excluded. The responses of 112 cells met the criterion for further data analyses, including 103, 20, 12, and 14 cells in Experiments 1, 2, 3, and 4, respectively. Some cells were tested in more than one of the four experiments.

**Figure 2:**
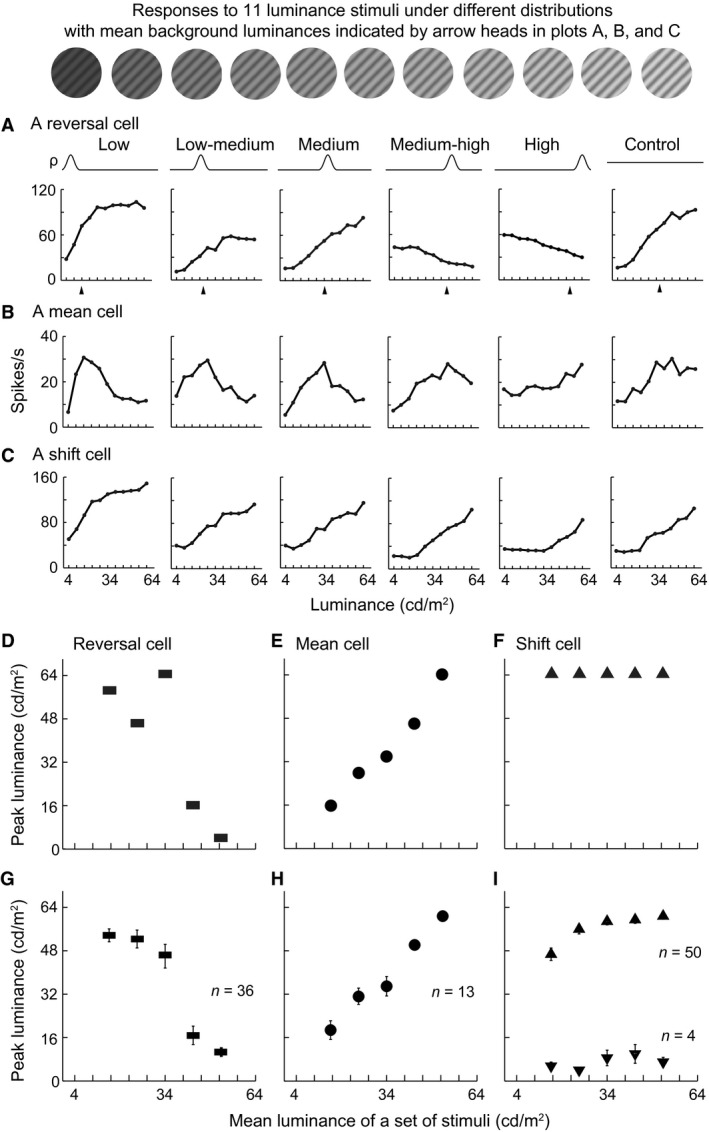
Dynamic responses to different luminance distributions in V1. (A–C) Examples of raw responses of reversal cells (A), mean cells (B), and shift cells (C) to six sets of luminance distributions in Figure 1B. All three cells were complex cells. *ρ* (probability) and curve represent a luminance distribution as in Figure 1 and correspond to the response curve elicited by the distribution. At the top, the 11 luminance stimuli from responses to which these luminance response function curves were extracted were shown. All stimulus sets contained the 11 stimuli in the study. (D–I) Relationship between the peak luminance (elicited the maximal response) of reversal cells (D, G), mean cells (E, H), and shift cells (F, I) and the mean luminance of stimulus distribution. The plots in (D), (E), and (F) were for the cells in (A), (B), and (C). The plots in (G), (H), and (I) were the averaged data for the populations of reversal cells, mean cells, and shift cells. In (H), linear regression, slope* *= 1.107, *r *=* *0.988, *P *=* *0.017. Note that 50 of the 54 shift cells preferred luminance increments (top of [I]), while 4 preferred decrements (bottom of [I]). Vertical bar: SEM.

**Figure 3:**
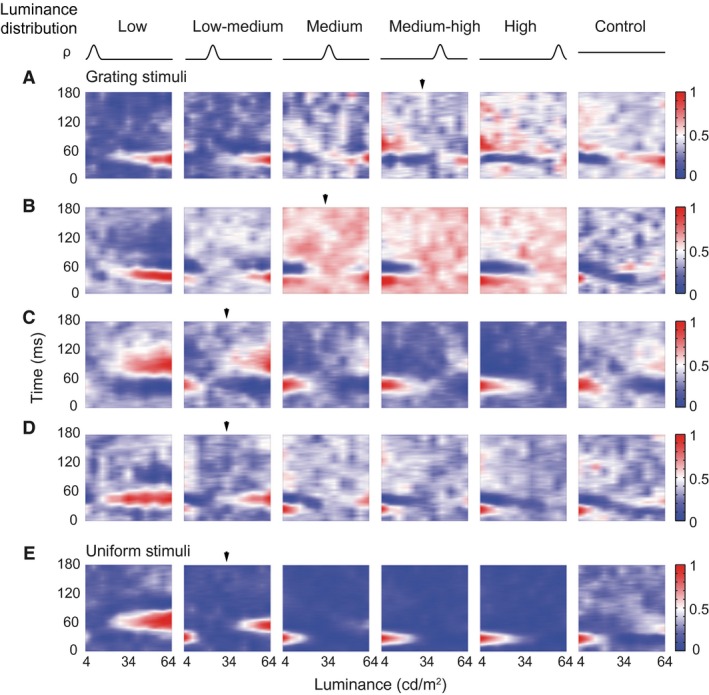
Reversal cells respond to both luminance decrements and increments when the high density of stimuli (HDS) is distributed in an intermediate range of luminance. (A–D) Four examples of reversal cells that responded to both luminance increments and decrements when the HDS was located in medium–high (A), medium (B), and low–medium ranges of luminance (C, D), as indicated by arrowheads. The reversal occurred at different time latencies (A, C, D) or the same latency (B) after stimulus onset (*y*‐axis of each panel). *x*‐axis was the luminance of stimuli. Blue‐white‐red color code: normalized responses. All four cells were complex cells. (D, E) Example responses of a reversal cell to luminance distributions expressed by gratings (D) and uniform patches (E). This cell exhibited similar response patterns to these two types of stimuli that had the same luminance distribution probabilities (*ρ*), luminance ranges, and stimulus sizes.

The responses of each cell to the experimental and control stimuli were sorted using a reverse correlation algorithm (Jones and Palmer 1987; DeAngelis et al. 1993; Ringach et al. 1997; Mazer et al. 2002; Nishimoto et al. 2005; Hu et al. 2011; Li and Wang 2013). We focused on analyzing the responses to luminance stimuli in steps of 6 cd m^−2^ covering the range from 4 to 64 (4, 10, 16, 22, 28, 34, 40, 46, 52, 58, and 64 cd m^−2^, see 11 stimuli at the top of Fig. 2). All stimulus sets in Experiments 1–4 contained the 11 stimuli. The response variance curves to these 11 stimuli were calculated from −300 to 180 msec in 1 msec step. The response variance during −300 to 0 msec before stimulus onset was estimated as the baseline level of responses. We calculated the mean and SD of the variances of responses from −300 to 0 msec. The mean + 5 SDs of the variances of the noise level was used as the criterion for the threshold of effective responses. We considered the responses of the cell as effective or significant when the variance of responses to different luminance stimuli after stimulus onset exceeded this threshold. The luminance response function (LRF) was calculated from the responses to the 11 luminance stimuli within a 20‐msec window centered at the peak of responses, which was the duration when the cell showed the maximal responses to luminance decrements or increments. The LRF was fitted with the Naka‐Rushton equation:

(2) $$R\left(L\right)=R_{\max}\cdot \left(L^{n}/\left(L^{n}+L_{50}^{n}\right)\right)+Baseline$$

as had been performed for the contrast response function (Albrecht and Hamilton 1982; Hu et al. 2011). Here, the *R*_max_ is the maximal response of LRF, the *L*_50_ is the luminance that evoked 50% of *R*_max_, the *Baseline* is the baseline activity of a cell regardless of the luminance changes during presentation of set stimuli, and *n* is the exponent that reflects the rate of response change or the slope of the function. The parameters of *L*_50_, *R*_max_, and *baseline* were extracted for the further analyses. Values of *n* were not observed to change consistently across different conditions and are not shown. Figure 6 illustrates the fittings for increment and decrement LRFs. For the decrement LRF, *R*_max_ was negative after data fitting with the equation. Because it is not possible for *R*_max_ to be negative, we used its absolute value. For those reversal cells that had two significant response peaks at different latencies in some conditions, we took the peak with the largest response magnitude for these analyses (e.g., some panels of Fig. 3). The value of adjusted *R* ^2^ was computed to evaluate how well the LRF is fit. Only the cells that had good LRF fit with adjusted *R* ^2^ >0.75 in this test over 400 repetitions were included in the analyses.

For the data in Figure 8H, the LRFs of a cell to each of 40 repetitions during L4 (or H4) presentation in the fourth experiment were averaged across 10 recycles of the switch presentation between the L4 and H4 stimulus sets. Then, we calculated the cumulative LRFs from the 1st repetition to 40th one and fitted these LRFs with the Naka‐Rushton equation to find the first cumulative LRF that had good fit with adjusted *R* ^2^ >0.75 during the 40 repetitions. The number of stimulus repetitions across which the first cumulative LRF occurred, that is, exhibited good tuning to luminance changes (adjusted *R* ^2^ >0.75), was regarded as the number of stimulus presentations that was necessary for the cell to establish stable response tuning. Therefore, the number was regarded as the time over which a cell was able to establish good tuning to luminance changes when the ambient luminance changed. Most cells could tune well to both L4 and H4 presentations before 40 repetitions, whereas some cells did not tune to either the L4 or H4 presentation when 40 repetitions had been finished. Cells that did not show good response tuning to both L4 and H4 presentation in the 40 repetitions were not included in the analysis.

#### Analyses for RF properties

Cells were classified as simple cells and complex cells according to the F_1_/F_0_ index of their modulated responses to drifting sinusoidal gratings (Skottun et al. 1991) at 100% contrast (Crowder et al. 2007). The RF spatiotemporal structures of these cells were mapped with briefly flashed sparse ON and OFF stimuli and analyzed with the reverse correlation method (Jones and Palmer 1987; DeAngelis et al. 1993; Mata and Ringach 2005; Malone et al. 2007; Dai and Wang 2012). A spatial overlap index (SOI) of the RF ON and OFF subregions (Mata and Ringach 2005; Dai and Wang 2012) was calculated by the following equation:

(3) $$\text{SOI}=\frac{(W_{\mathrm{ON}}+W_{\mathrm{OFF}})/2-\text{Sep}}{(W_{\mathrm{ON}}+W_{\mathrm{OFF}})/2+\text{Sep}}$$

The spatial profiles at *x* dimension of the RF ON and OFF subregions were obtained from the variance curve of the responses at the optimal time using a reverse correlation algorithm and were fitted by a Gaussian function. The *W*_ON_ and *W*_OFF_ were the widths (degrees) of the ON and OFF subregions, respectively, at the 25% height of the fitted spatial curve. Sep (degrees) was the separation between the centers of the ON and OFF subregions. [(*W*_ON_ + *W*_OFF_)/2 + Sep] was the overall RF width, and [(*W*_ON_ + *W*_OFF_)/2 − Sep] was the overlap zone between the ON and OFF subregions. Thus, the SOI was the ratio of the overlap zone to the total RF width. The SOI ranged from a negative value to 1, which indicates that the ON and OFF subregions ranged from segregation to complete overlap.

Moreover, we quantitatively compared the strength of the RF ON response with the OFF response for these cells. The response strength was defined as the ratio of signal to noise according to the spatial energy (or variance) of the responses (Malone et al. 2007; Yeh et al. 2009). The signal was the mean value of the variances (20 data points) during 20 msec centered at the peak of the variance curve of the responses to the sparse ON or OFF stimuli applied to map the RF of a cell above. The noise was the mean value of the variances during −150 to 0 msec prior to stimulus onset (150 data points). When the relative strength (signal/noise ratio, SNR, of an ON or OFF response) was >2, the cell responses were considered significant. Only the cells with a significant ON and/or OFF response during 0 to 100 msec after stimulus onset were included in the analysis. After the SNRs of the ON and OFF responses were obtained, the relative strength between the ON and OFF responses was evaluated by an ON–OFF index:

(4) $$\text{ON}-\text{OFF}\;\text{index}=\frac{{\text{SNR}}_{\mathrm{ON}}-{\text{SNR}}_{\mathrm{OFF}}}{{\text{SNR}}_{\mathrm{ON}}+{\text{SNR}}_{\mathrm{OFF}}}$$

where the SNR_ON_ and SNR_OFF_ were the SNRs of the ON and OFF responses. The ON–OFF index ranged from −1 to 1. A positive (or negative) ON–OFF index indicates that the ON response is stronger (or weaker) than the OFF response.

The RF size of the cells was the width measured at 25% of the peak magnitude of the spatial profile along the *x* dimension of the RF across the entire RF, including both the ON and OFF subregions (Dai and Wang 2012). The TF and SF tuning curves were fitted by a log‐Gaussian, and the preferred TFs and SFs were extracted from the fitted curves (Li and Wang 2013). The strength of the orientation selectivity was evaluated via the circular variance (CV), a measure for the global orientation tuning (Dai and Wang 2012). The CV is highly robust against variations in the data derived from noises. The value of the CV ranges from 0 (high) to 1 (low orientation selectivity).

## Results

To simulate rapid luminance changes, a set of sinusoidal gratings with the same contrast, orientation, spatial frequency, and size (five times in diameter >RF) but different luminance levels (49 stimuli from 4 to 64 cd m^−2^) was presented to the RF of a V1 neuron on a background corresponding to the average luminance of all grating stimuli in the set. The 4–64 cd m^−2^ of luminance was in the typical range of natural images (Frazor and Geisler 2006). The contrast of all gratings was 70% to avoid neuronal responses saturation by 100% contrast. Each set of stimuli contained a HDS (83.6%, 41/49) that were distributed in a narrow range of luminance, and the remaining 16.4% (8/49) were uniformly distributed in the remaining luminance range. Five different stimulus sets were used. The HDS was concentrated in different local ranges (top of Fig. 1B). The control condition consisted of 49 luminance stimuli that were uniformly distributed across the 4–64 cd m^−2^ range. Stimuli in a set were flashed randomly at 50 Hz without intervals in a block (Li and Wang 2013). This yielded a consecutive and random sequence of luminance changes over time (Fig. 1A). When different stimulus sets were applied to a V1 neuron, the mean value of luminance distribution (and therefore the background luminance) also changed (Fig. 1A) because the different stimulus sets had HDSs located within different local ranges of luminance (Fig. 1B).

### Different response behaviors of V1 cells to change of luminance distribution

**Figure 4:**
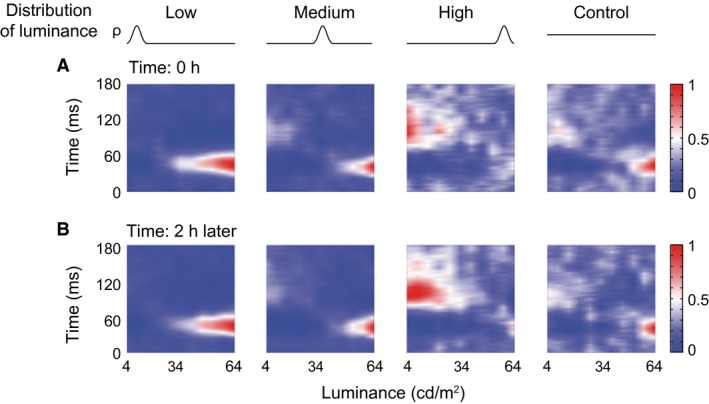
Repeatability of responses of reversal cells to different luminance distributions. The reversal response pattern of a cell (complex cell) (A) was replicated after 2 h (B) using the same conditions (*ρ* curves) of high density of stimuli distributed in low, medium, and high ranges of luminance and a uniform distribution condition (Control).

Neurons were sorted into three groups according to the decrease or increase profile (negative or positive slope) of the LRF with different luminance distributions and the luminance that evoked the peak response (peak luminance). The first group of cells exhibited a decreased LRF and peak response to the lowest luminance under a high luminance distribution and an increased LRF and peak response to highest luminance under a low luminance distribution (Fig. 2A). Among the 103 V1 cells that exhibited significant responses, 35% of the cells (*n* = 36) were in the first group. The cells reversed the increasing LRF to decreasing when HDS changed from a low to high luminance range and reversed the peak response from luminance increments to decrements. For example, the cell shown in Figure 2A responded maximally to luminance increments when the HDS was located in low (4–16 cd m^−2^), low–medium (16–28 cd m^−2^), and medium (28–40 cd m^−2^) luminance ranges, whereas it responded maximally to luminance decrements when HDS was located in the medium–high (40–52 cd m^−2^) and high (52–64 cd m^−2^) luminance ranges. This response reversal pattern is revealed by plotting the peak luminances against the mean luminances of distributions (Fig. 2D) and characterized by a large drop in the peak luminance (from 64 to 16 cd m^−2^; *y*‐axis of Fig. 2D). The averaged data from the group of 36 cells further supported the observation (Fig. 2G). For many of these cells, the reversal occurred between the medium and medium–high luminance ranges (Fig. 2A, D, G). Furthermore, these reversal cells responded to both luminance decrements and increments when HDS was distributed in a certain middle range of luminance (Fig. 3). The reversal of response profile was not a stochastic fluctuation in neuronal activity and was repeatable (Fig. 4). Reversal responsiveness was also consistently observed in the cells tested with uniform patches of surface luminance stimuli (Fig. 3E).

The second group of cells (13/103; 13%) had an LRF profile analogous to an upside‐down V shape and a peak response to stimuli distributed around the mean luminance of each of the five stimulus sets. That is, the peak of their LRFs gradually shifted as the position of HDS was changed from low to high luminance range (Fig. 2B). Their LRFs were not monotonically increasing or decreasing. The peak luminance of these cells increased linearly with the mean luminance of different HDS distributions (e.g., Fig. 2E). The linear correlation was significant in the population of 13 cells (*r *=* *0.99, *P *=* *0.017; Fig. 2H).

The remaining group of cells (54/103; 52%) exhibited increasing (Fig. 2C) or decreasing LRFs across all HDS distributions. Usually the peak luminance of these cells did not change with luminance distribution (e.g., Fig. 2F). Although a few of cells did not respond maximally to the highest luminance of 64 cd m^−2^ in low and low–medium luminance distribution (triangles in upper left of Fig. 2I), their LRF profiles were increasing under the two conditions. Thus, the LRF profile shapes and peak responses of these cells did not largely change with the variation in the luminance distribution. Their LRFs displayed a systematic rightward shift, which resembled the adaptive responses of V1 cells to contrast (Ohzawa et al. 1982; Bonds 1991; Hu et al. 2011). Of the 54 cells, 50 had the largest responses to luminance increments (top of Fig. 2I) with increasing LRF profiles (Fig. 2C) across all conditions, whereas the other four had the largest responses to luminance decrements (bottom of Fig. 2I) with decreasing LRF profiles (not shown).

### Neurons were sensitive to intensity of luminances distributed in a narrow range

**Figure 5:**
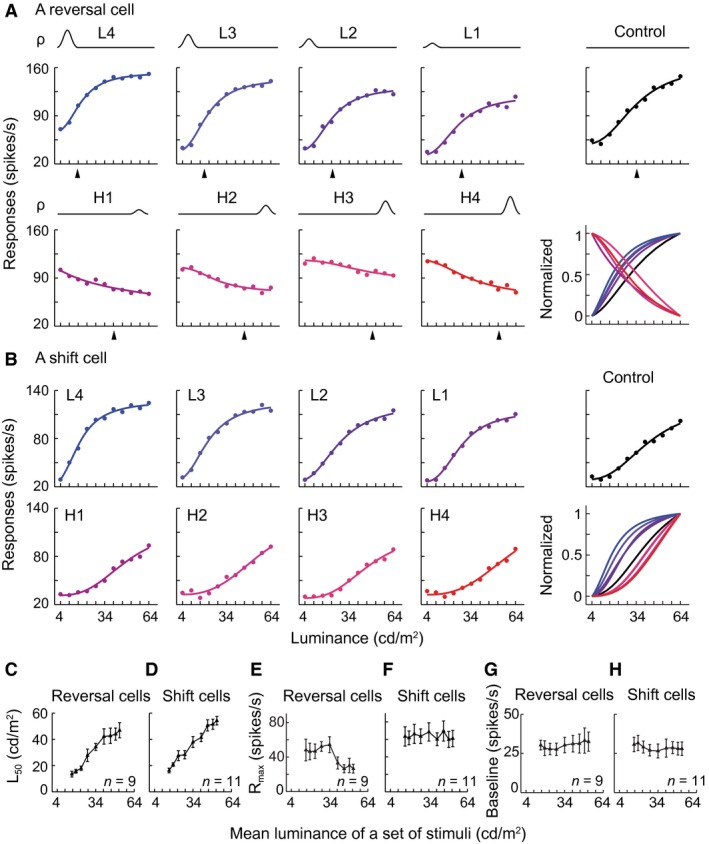
Luminance response functions (LRF) were systematically modulated by the intensity of the luminance distribution. (A, B) Representative responses of a reversal cell (complex cell) and a shift cell (complex cell) to nine sets of luminance distributions in Figure 1C. Dots represent raw data calculated from a reverse correlation algorithm. Curves were fitted by the Naka‐Rushton equation. In the lower right panels of (A, B), each curve was normalized by the largest value minus the smallest value of a fitted LRF. A color indicates the corresponding LRF and normalized curve. (C–H) Effects of the gradual changes of luminance distribution on the *L*_50_, *R*_max_, and *baseline* of the LRFs of reversal cells (*n* = 9) and shift cells (*n* = 11). In (C), linear regression, slope = 0.95, *R* ^2^ = 0.96; *P *<* *0.01. In (D), slope = 1.01, *R* ^2^ = 0.98; *P *<* *0.01. In (E), for each of the left 4 data points compared with the control (central point), *t*‐test, all *P *>* *0.05, *n* = 9; and for each of the right 4 data points compared with the control, all *P *<* *0.05, *n* = 9. In (F–H), one‐way ANOVA for each of the three cases, all *P *>* *0.05; *n* = 11 cells, *n* = 9 cells, and *n* = 11 cells). The center data points of (C–H) were the responses to the control distribution. Arrowheads: mean luminance of a stimulus set. Vertical bar: SEM.

The previously described response variations were due to the change in the luminance range in which HDS were distributed. Therefore, the LRF of a cell may gradually change when the density of HDS within a specific range increases. If this is true, the HDS is critical in the induction of the response changes of a neuron under different luminance distributions. This hypothesis was tested using eight experimental stimulus sets, in which the density of HDS (and therefore the average luminance) was systematically changed in low or high luminance ranges, as well as the control set of uniform distribution (Fig. 1C). For example, in the low luminance range (4–16 cd m^−2^), as the stimulus number increased from 3 of the control condition to 7, 13, 23, and 41 of the HDS distributions, the LRF of a reversal cell gradually shifted leftward (L1–L4 panels of Fig. 5A). In contrast, as the stimulus number increased in the high luminance range (52–64 cd m^−2^), this cell switched its LRF from an increment profile (Control panel: 3 stimuli) to a decrement profile (H1 panel of Fig. 5A: 7 stimuli), and its LRF gradually shifted rightward (H1, H2, H3, and H4 panels: 7, 13, 23, and 41 stimuli, respectively). Only 4 additional stimuli produced the dramatic switch in the LRF profile (compare Control and H1 panels in Fig. 5A). This cell was highly sensitive to the density change in HDS of the luminance distribution. Figure 5A indicates that the reversal in the response preference for the luminance increments and decrements presented in Figure 2A was not an artifact. For shift cells, increasing the density of the HDS distributed in the low luminance range caused a leftward shift of LRF (panels L1–L4 of Fig. 5B), whereas increasing the density in the high luminance range caused a rightward shift of LRF (panels H1–H4 of Fig. 5B). This can be seen in the plots of the normalized LRFs in the bottom right panel of Figure 5B. The data in Figure 5B also confirmed that the change in the luminance distribution caused the LRF shift of the shift cell shown in Figure 2C.

**Figure 6:**
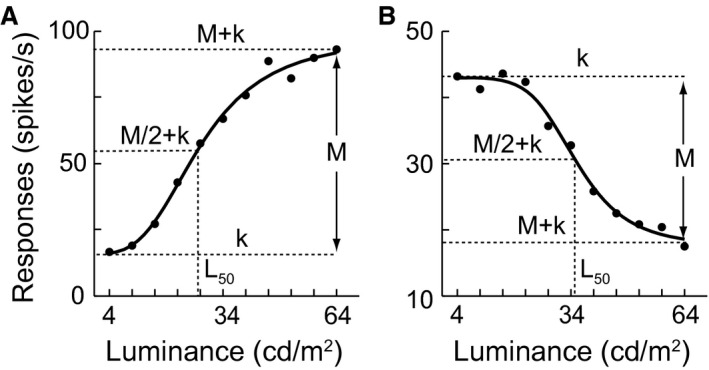
Graphic illustration for fitting luminance response functions (LRFs) of V1 cells. (A) An increment LRF profile. (B) A decrement LRF profile. Each LRF was fitted with the Naka‐Rushton equation to evaluate the modulated effects of changes in the luminance distribution on neuronal responses. Conventionally, the Naka‐Rushton equation {*R*(*L*) = *R*_max_·[*L* ^*n*^/(*L* ^*n*^ **+ *L*_50_^*n*^)] + *baseline*} well describes the response of V1 cells to contrast. For the parameters of *R*_max_, *L*_50_, *baseline*, and *n*, see Data analyses. To fit both the increment and decrement LRFs, we defined *M* (magnitude) as the *R*_max_ and *k* as the *baseline* of the LRF using the Naka‐Rushton equation {*R*(*L*) = *M*·[*L* ^*n*^/(*L* ^*n*^ **+ *L*_50_^*n*^)] + *k*}. Thus, for an increment curve, *R*_max_ = *M* and *baseline *= *k* as shown in (A), and for a decrement curve, *R*_max_ = |*M*| and *baseline *= *M *+ *k* as shown in (B). Note that *M* is negative for a decrement curve.

The LRFs were fitted with Naka‐Rushton eq. (2) (Fig. 6). Quantitative analysis of these results demonstrated that the luminance gain (*L*_50_) of LRF observed in both reversal cells and shift cells was modulated systematically by changes of HDS intensity (quantified as the change in the average luminance; Fig. 5C, D). The *L*_50_ is the luminance that elicited 50% of the maximal response (*R*_max_). The firing rate of a neuron is most sensitive to luminance changes around *L*_50_. When the mean luminance changed from 15.4 cd m^−2^ of the L4 distribution to 34 cd m^−2^ of the uniform (control) distribution and then to 52.6 cd m^−2^ of the H4 distribution, *L*_50_ of both groups of cells consistently increased (Fig. 5C: *R* ^2^ = 0.96, *P *<* *0.01; Fig. 5D: *R* ^2^ = 0.98, *P *<* *0.01). For reversal cells, the response gain (*R*_max_) to HDS increase in the low luminance range was larger than that in the high luminance range (Fig. 5E). This illustrates that reversal cells decreased the response magnitudes in the high luminance range compared with the low luminance range. The *baseline* responses were not significantly different between the two distributions (Fig. 5G). For shift cells, *R*_max_ and *baseline* values were not significantly different between the low and high luminance distributions (Fig. 5F, H), respectively, and were also not significantly different from that in the uniform (control) distribution. Note that the center data point of each panel of Figure 5C–H is the value from the uniform distribution experiment (control panels of Fig. 5A, B).

### Neurons were sensitive to asymmetry of luminance distribution

**Figure 7:**
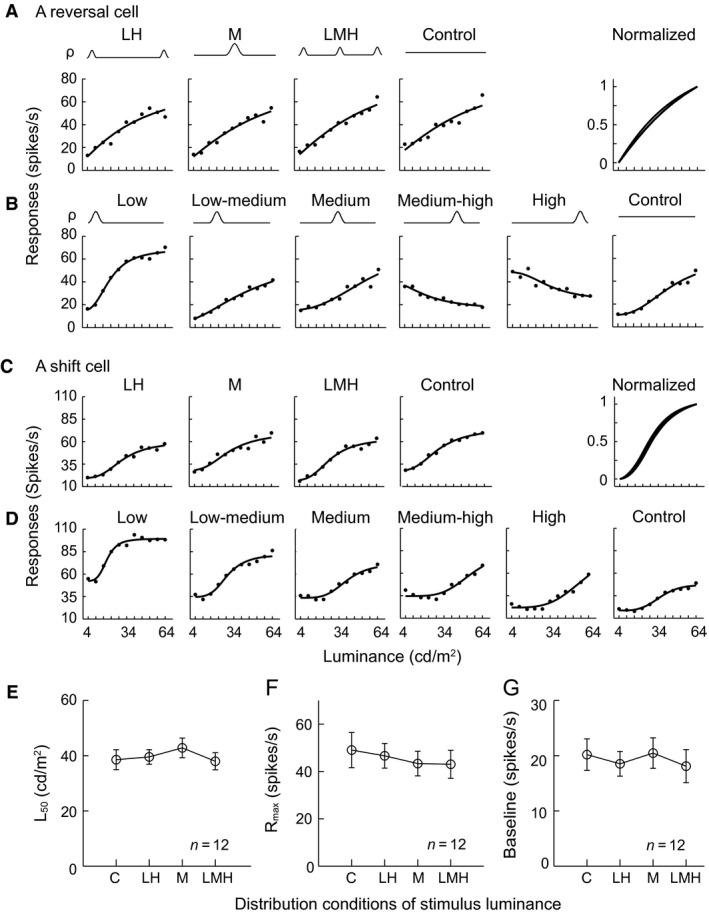
Symmetrical distribution of luminance does not cause a dramatic reversal or systematic shift of the luminance response function (LRF). (A, C) Example responses of a reversal cell (simple cell) and a shift cell (complex cell) to the four sets of symmetrical luminance distributions in Figure 1D. (B, D) Responses of the reverse cell and shift cell to the six sets of stimuli in Figure 1B. In (A) or (C), the nearly complete overlap of the normalized curves of the LRFs in the upper right panels indicated that these LRFs were very similar to one another in the reverse cells or shift cells responding to the symmetrical luminance distributions (LH: low–high; M: medium; LMH: low–medium–high; Control). In (B) or (D), the response behaviors to the luminance distributions in Experiment 1 were similar to the behaviors indicated in Figure 2A for reverse cells or Figure 2C for shift cells. (E–G) Different symmetrical distributions of luminance had no effects on the *L*_50_, *R*_max_, or *baseline* of the LRFs of the reversal cells or shift cells. Vertical bar: SEM. In (E–G), one‐way ANOVA for each of the three cases, all *P *>* *0.05.

The response pattern elicited in response to the medium HDS distribution was often similar to that observed in response to the uniform distribution (control condition) that was of symmetry (e.g., Figs. 2A, C, 3, 4). This raised the question of whether the changes in response to different luminance distributions were related to the asymmetry of the luminance distributions. This possibility was tested using three stimulus sets with HDSs that were distributed symmetrically but in different luminance ranges, plus the uniform control condition (Fig. 1D). Reversal cells had similar LRFs across all four conditions (Fig. 7A). In contrast, they reversed the LRFs from low to high luminance distributions in Experiment 1, which contained asymmetrical distributions (Fig. 7B). The shift cells also had similar LRFs across the four conditions (Fig. 7C); however, they shifted the LRFs from low to high luminance distributions in Experiment 1 (Fig. 7D). The *L*_50_, *R*_max_, and *baseline* values of all tested reversal and shift cells (*n* = 12 cells) were not significantly different across the four symmetrical conditions (Fig. 7E–G). Therefore, the asymmetry of luminance distribution, relative to the mean luminance, is crucial to the observed considerable changes or modulations in response.

### Response changes to luminance distributions were not derived from slow changes of responses over time

**Figure 8:**
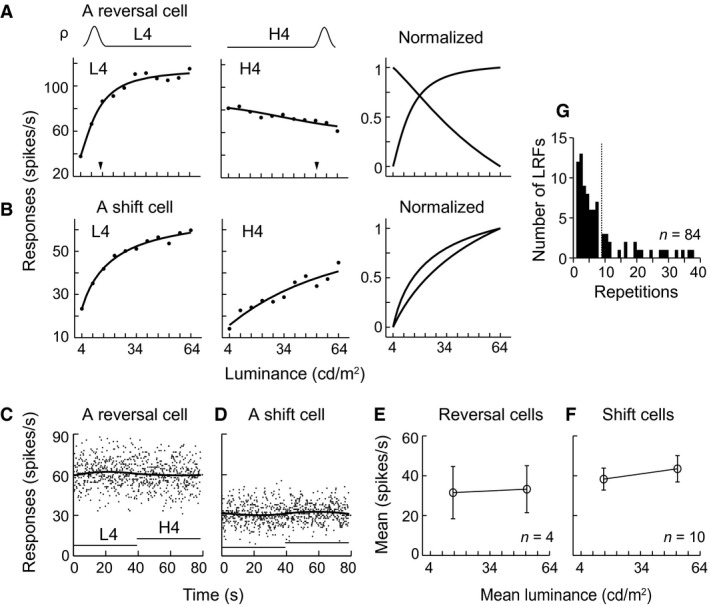
Distinct responses and fast adaptation of V1 cells to switching presentation between different luminance distributions over time. (A, B) Example responses of a reversal cell (complex cell) and a shift cell (complex cells) with stable firing rates (C, D) showed distinct response profiles to switching stimulation shown in Figure 1E. (C, D) Mean firing rates of the two example cells in (A, B) was stable over time. Dots: raster responses (bin = 100 msec) during 78.4 sec. Dark curves: firing rates averaged from 10 cycles (switches). Horizontal line: 39.2 sec during which L4 or H4 was presented for 40 repetitions. The level of horizontal lines indicates that high density of stimuli was distributed in low (L4) or high (H4) luminance ranges. (E–F) Mean firing rates of 4 reversal cells and 10 shift cells during L4 and H4 presentations. Vertical bar: SEM. (G) Distribution of the numbers of stimulus presentations (repetitions) across which LRFs of cells achieved stable tuning to L4 and/or H4 sets of stimuli. Dashed line: mean value, 8.9 ± 8.89 (SD) repetitions, *n* = 84 LRFs (from 49 cells).

One might argue that the observed results reflect slow changes in the response magnitude because responses to different HDS conditions were acquired in separate temporal blocks. Here, we demonstrate that these results were not due to the stimulus block design. We tested 49 cells by alternately presenting the two stimulus sets, one with HDS distributed in the low luminance range and the other in the high luminance range (L4 and H4 of Fig. 1E, i.e., L4 and H4 of Fig. 1C), in one block (right panel of Fig. 1E). In the test, after the L4 (or H4) set of stimuli was presented for 40 repetitions, the stimulation was switched to presenting the H4 (or L4) set of stimuli, respectively, for 40 repetitions. The switch process was repeated continuously for 10 cycles (Figs. 1E, 8C, D). We focused this analysis on the cells (*n* = 14) without obvious fluctuations in their averaged responses to the two sets of stimuli over time (Fig. 8C, D). At first glance, the flat average responses seemed to contain no information about stimulus luminance (Fig. 8C–F), but they actually did (Fig. 8A, B). First, reversal cells had increment and decrement LRFs (Fig. 8A), while shift cells had distinct increment LRFs (Fig. 8B). Second, these LRFs were similar to those observed when cells were tested with these two sets of stimuli presented in separated blocks in Experiment 2 (comparing the L4 and H4 panels of Fig. 5A, B with those in Fig. 8A, B). These observations illustrated that the flat average responses over time (Fig. 8C–F) contained distinct LRFs (Fig. 8A, B). Therefore, the response changes cannot be explained by the slow changes or fluctuations in the response magnitude of V1 neurons over time, and they are therefore dependent on the changes in the statistics of luminance distribution.

### Time course of responses in adapting to luminance distribution

Having demonstrated that the V1 neurons changed their LRFs in accordance to changes in the distribution of luminance, it was important to determine how long it takes for a cell to adapt to a change in luminance distribution. To this end, we determined how many stimulus representations were necessary for a cell to achieve stable response tuning to a change in luminance distribution. We examined the time course of LRFs of the cells to L4 and H4 sets of stimuli that were tested in the switch experiment above (Figs. 1E, 8A–F). Figure 8G shows the distribution of the numbers of stimulus repetitions (trials or presentations) required for a cell to achieve good tuning (LRF with adjusted *R* ^2^ >0.75 as the criterion for a good fit) to L4 and H4 sets of stimuli during 40 repetitions (Figs. 1E, 8C, D). For most cells, this number was distributed in the early part of the 40 repetition (39.2 sec). The mean value of the distribution is 8.9 repetitions (8.72 sec). This means that on average these cells could tune well to luminance changes in 8.72 sec. Approximately 30% of the LRFs (25/84) were well tuned to luminance changes by 3 repetitions (2.94 sec), and 73% (61/84) were well tuned by 8 repetitions (7.84 sec) in the available 84 LRFs of 49 cells. Thus, most cells are capable of establishing a stable tuning response to luminance changes within several repetitions after the luminance distribution changes, and the co‐activities of a group of cells can fast detect the sudden changes of ambient luminance.

### RF properties of reversal cells, shift cells, and mean cells

**Figure 9:**
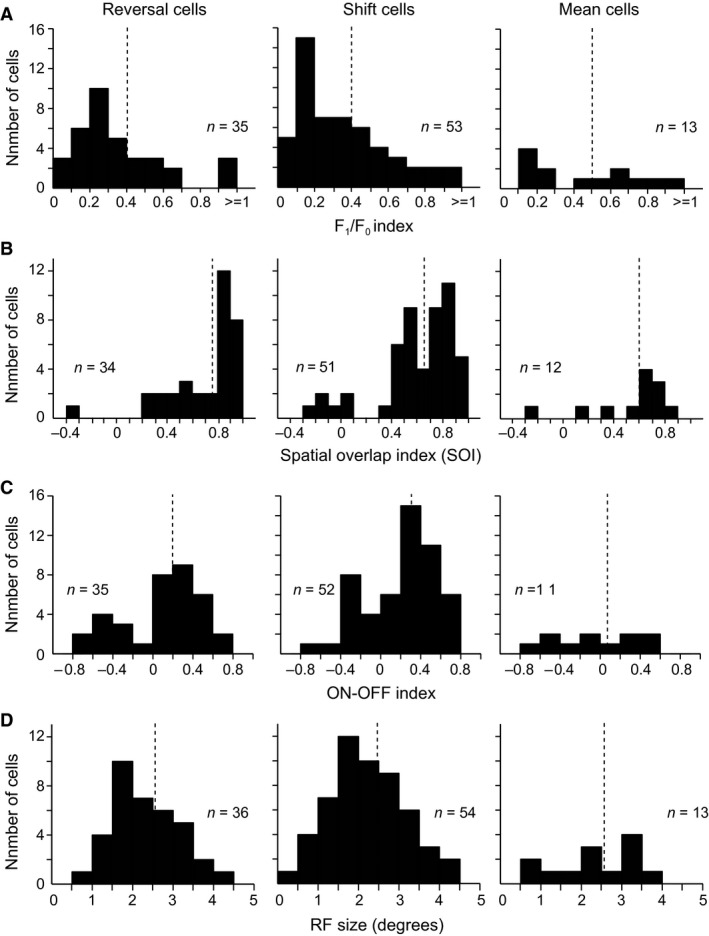
Distributions of F_1_/F_0_ index, SOI, ON–OFF index and receptive field (RF) size of reversal cells, shift cells, and mean cells. Histograms of the F_1_/F_0_ index of the responses to drifting gratings (A), the spatial overlap index (SOI) of the RF ON and OFF subregions (B), the ON–OFF index of the RF ON and OFF responses (C), and the size of the RF (D) for reversal cells, shift cells, and mean cells. Cells with F_1_/F_0_ > 1 were simple cells and those with F1/F0 < 1 were complex cells in (A). Cells with SOI < 0.3 were simple cells and those with SOI > 0.5 were complex cells in (B). *n*: number of cells available for each of these analyses. The dashed lines indicate the mean values.

Next, we investigated whether the three groups of reversal, shift, and mean cells were different in the frequencies of simple and complex cells and the RF spatial organization. Simple and complex cells were identified by the F_1_/F_0_ index of their modulated responses to 100% contrast sinusoidal gratings drifting at their preferred direction. In our sample, 5.9% of the cells (6/101) were simple cells. The distributions of the F_1_/F_0_ indices were similar in the three groups (Fig. 9A). The percentages of the simple cells in the reversal cells (8.6%, 3/35), shift cells (3.8%, 2/53), and mean cells (7.7%, 1/13) were not significantly different (all three pairwise comparisons, *P *>* *0.05, *t*‐test), although this might be due to the small number of simple cells. Consistently, the distributions of the spatial overlap index (SOI) of RF ON and OFF subregions were not significantly different in the three groups (Fig. 9B). The mean SOIs of the reversal cells (0.76 ± 0.29 (SD), *n* = 34), shift cells (0.66 ± 0.3, *n* = 51), and mean cells (0.6 ± 0.32, *n* = 12) were similar (all three *P *>* *0.05, *t*‐test). Only the cells that had both ON and OFF subregions were included in the SOI analysis. The percentages of the cells that had a sole ON or OFF subregion were 5.6% (2/36), 5.6% (3/54), and 7.7% (1/13) in the reversal cells, shift cells, and mean cells, respectively, without significant differences. Thus, these cells had no distinction in the classification of simple and complex cells measured with drifting gratings and the spatial RF structure measured with sparse ON and OFF stimuli. We also compared the ON and OFF response strengths of these cells using an ON–OFF index. A value of the ON–OFF index >0 (or <0) indicates that ON response is larger (or smaller) than OFF response. The ON–OFF index distribution of reversal cells tended to be bimodal. Most shift cells had ON responses stronger than OFF responses. The ON–OFF indices of mean cells distributed flatly (Fig. 9C). Furthermore, reversal cells, shift cells, and mean cells were not different in the RF size measured with sparse stimuli (Fig. 9D). Additional analyses indicate that the three groups of cells were not different in the preferred TF, SF, or orientation selectivity strength (Table 1).

**Table 1:** Temporal frequencies (TF), spatial frequencies (SF), and orientation selectivity strength (CV) of mean cells, reversal cells, and shift cells

	TF (mean ± SD)	SF (mean ± SD)	CV (mean ± SD)
Reversal cells	5.29 ± 2.48^1^ (*n* = 28)	0.70 ± 0.32^2^ (*n* = 36)	0.70 ± 0.14^3^ (*n* = 36)
Shift cells	4.51 ± 1.77^1^ (*n* = 42)	0.73 ± 0.42^2^ (*n* = 54)	0.67 ± 0.14^3^ (*n* = 54)
Mean cells	4.60 ± 2.39^1^ (*n* = 12)	0.79 ± 0.25^2^ (*n* = 13)	0.57 ± 0.16^3^ (*n* = 13)

^1,2,3^For all pairwise comparisons, *P *>* *0.05, *t*‐test. *n*: number of cells, only the cells available for each analysis were included. CV: circular variance, its value ranges from 0 (high) to 1 (low orientation selectivity).

We further examined whether the nearby cells exhibited similar response behaviors by checking the penetrations along which three or more cells were consecutively recorded, and the recording distance was more than 300 *μ*m. If the consecutively recorded cells in this distance exhibited the same reversal, shift, or mean type of response behavior, the data were included in the analysis. The data from 15 cells of 4 penetrations indicated that the shift group cells clustered together in the distance of 470 ± 169 *μ*m (*n* = 4 penetrations). The longest distance was 690 *μ*m where 6 shift cells were recorded. Because the recording for each cell lasted for 2–4 h in the preliminary test for stimulus parameters and in Experiment 1 and even longer for some cells that were further tested in Experiments 2–4, we could not record enough cells in a penetration to obtain sufficient data to more definitely address the issue, and we did not have available data for the mean or reversal cells.

## Discussion

Our study shows that V1 cells encode luminance distribution in three different ways. The intensive distribution of luminance in a narrow range and the luminance distribution asymmetry are two critical factors in determining the responses of V1 cells to luminance distributions. Many cells may establish stable luminance tuning in several seconds following luminance distribution changes. Mean cells represent the prevailing luminance in the current environment. Reversal cells represent the salient luminance changes (decrements and increments) relative to the prevailing mean luminance. Shift cells effectively discriminate luminance changes by shifting their dynamic responses according to the prevailing luminance. The change in the luminance gain (*L*_50_) parameter of the LRF is a good indicator of the response variation of neurons to the change in luminance distribution. For both reversal and shift cells, the luminance gain increases with the increase in the mean luminance of the stimulus distribution (Fig. 5C, D). This indicates that the luminance to which a neuron is most sensitive shifts from a low to high range when the prevailing luminance of a distribution increases. For mean cells, the luminance that evokes the peak response also increases with the increase in the mean value of a luminance distribution (Fig. 2E, H). Thus, all three groups of cells are sensitive to luminance distribution change by dynamically adjusting their responses in different modes. These findings indicate that V1 neurons detect luminance changes according to the luminance distribution in the current environment. The reversal and mean cells are more important than shift cells in representing luminance distribution change.

### Luminance response in V1

Responses of V1 cells to luminance changes have been investigated with diffuse light that was illuminated for seconds to minutes (Bartlett and Doty 1974), uniform luminance stimuli that were statically presented for 500 msec with an interstimulus interval (Maguire and Baizer 1982; Kinoshita and Komatsu 2001; Huang and Paradiso 2008) or rapidly changing for 40 msec without a pause (Li and Wang 2013) or continuously changing in sine wave (Rossi et al. 1996; Peng and van Essen 2005; Dai and Wang 2012), contrast grating stimuli that were fast changing in luminance for 200 msec with a pause (Geisler et al. 2007) or 40 msec without a pause (Li and Wang 2013), and small sparse ON and OFF stimuli that were randomly flashed in space for 20 or 16 msec (Yeh et al. 2009; Komban et al. 2014). These studies indicate that V1 cells convey a substantial amount of luminance information regarding visual stimuli. Cells in V1 have also been demonstrated to represent the brightness of uniform surfaces (Rossi et al. 1996; Kinoshita and Komatsu 2001; Roe et al. 2005; Hung et al. 2007). However, no study has investigated whether V1 cells are sensitive to the luminance distribution. The current findings indicate that V1 cells also code the luminance distribution. The grating stimuli used are somewhat simpler than natural stimuli (Sharpee et al. 2004; Felsen and Dan 2005) however, they contained the richness of natural stimuli in the dimension of luminance change because the random presentation of stimulus luminances under different distributions simulates what occurs in natural vision (Dong and Atick 1995; Lesica et al. 2006). Under normal viewing conditions, the luminance information received by the eyes rapidly changes as the images on the retina vary with body, head, and eye movements; thus, the luminance within RF of individual V1 cells dynamically and rapidly varies with time. Through the dynamic luminance stimulation, we captured the response behaviors of three groups of V1 cells to transiently changing luminance with the changes in luminance distribution.

On the basis of the relationships of the LRF profile change and the peak luminance change with the luminance distribution change, V1 cells were distinguished into reversal cells, shift cells, and mean cells. However, the three groups of cells were not observed to have a clear difference in the analyzed RF response parameters (Fig. 9 and Table 1). This is understandable considering that the change in the luminance distribution is not the change in the stimulus feature dimension. The change in the distribution of a feature may only modulate responses of V1 cells, such as the responses that occur in the contrast distribution (Ohzawa et al. 1982; Hu et al. 2011). The gradual LRF shift of shift cells and mean cells are exactly the modulation effect. Reversal cells also exhibit the shift effect of LRF when we focus solely on the responses to luminance increments (or decrements) across different distribution conditions (Figs. 3, 4). The remarkable change of reversal cells from increasing to decreasing LRF profiles across different luminance distributions may be exception to the modulated effect because their responses to luminance increments and decrements are likely driven by ON and OFF responses, and the ON (or OFF) responses may be suppressed by the dense bright (or dark) stimuli in a high (or low) luminance distribution as subsequently discussed.

### Possible mechanisms for coding luminance distribution

In the early visual system from the retina through the LGN in the thalamus to V1, luminance information is transmitted along ON and OFF channels in which cells respond to luminance increments (ON) and decrements (OFF) (Kuffler 1953; Hubel and Wiesel 1961; Westheimer 2007; Balasubramanian and Sterling 2009). The ON (or OFF) cells in the retina and LGN converge the inputs of bright (or dark) luminance signals in a local field to an ON (or OFF) subregion of a cell in V1. The convergence or integration of luminance information forms the RF spatial structure of V1 cells composed of ON and OFF subregions. The elongated arrangement of the ON–OFF subregions generates the orientation selectivity of V1 cells and enhances their sensitivity to the contrast formed by the dark and bright luminances in their RFs (Hubel and Wiesel 1961; Reid and Alonso 1995; Jin et al. 2011; Wang et al. 2015; Kremkow et al. 2016; Lee et al. 2016). The V1 contains simple cells and complex cells. The ON and OFF subregions of simple cells are separate in space, whereas the subregions of complex cells overlap. It is possible that the distinctive responses of V1 cells to luminance increments and decrements under different luminance distributions relate to the ON–OFF structure of their RFs. However, we did not observe that the mean cells, reversal cells, and shift cells were clearly different in the classification of simple cells and complex cells (Fig. 9A) and in the RF spatial structure (Fig. 9B). This is likely due to the result that the number of simple cells was small in our sample. It is possible that differences would be identified if a reasonable percentage of simple cells was included. We measured F_1_/F_0_ using 100% contrast drifting gratings because only the cells with a modulation index (F_1_/F_0_) >1 in response to 100% contrast gratings can be regarded as real simple cells (Crowder et al. 2007). The low percentage of simple cells in our sample approximates to the previous results (Crowder et al. 2007; Dai and Wang 2012; Li and Wang 2013). More complex cells appear to be simple cells if F_1_/F_0_ is measured with low‐contrast gratings (Crowder et al. 2007). Moreover, the noncorrelation between RF ON and OFF structure and responses to luminance changes may be related to the fact that the stimuli we used to investigate the coding of V1 cells to the luminance changes occupied a large region of the visual field. The stimulus size was substantially larger than the RF size. In this circumstance, a cell must integrate luminance signals in a region substantially larger than its classical RF. Thus, the responses of a V1 cell to the luminance changes over a large region are different from the responses to the luminance changes that are presented sparsely by small ON and OFF stimuli within its RF. In this case, the luminance response property to large stimuli cannot be interpreted by the RF response properties measured with small sparse stimuli. In addition, the presentations of the sparse bright and dark stimuli are spatially segregated. These differences in stimulation may account for the observation that the response behaviors of V1 cells to luminance changes under different luminance distributions are not closely correlated with their RF structures.

Subcortical inputs have been suggested to contribute to the responses of cat V1 cells to luminance changes (Dai and Wang 2012; Li and Wang 2013; Wang et al. 2015). Because retinal photoreceptors and ganglion cells have shown rightward shift response curves to luminance increments with different background luminances (Sakmann and Creutzfeldt 1969; Boynton and Whitten 1970; Normann and Perlman 1979; Schneeweis and Schnapf 1999; Rieke and Rudd 2009), V1 shift cells probably inherit their shift responses from the counterparts of the early visual pathway. The observation that most shift cells have stronger responses to ON stimuli than to OFF stimuli seems consistent with the finding that they have increasing LRFs across all conditions of luminance distributions. For V1 reversal cells, the responses to luminance increments and decrements may be explained by the responses of ON and OFF channels of the early visual pathway. Retinal ganglion cells and LGN cells possess luminance‐dependent responses to full‐field step ON and OFF stimuli (Tikidji‐Hamburyan et al. 2015). These subcortical ON and OFF responses to ambient luminance may drive V1 cells responding to luminance increments and decrements. Under a low luminance distribution, the ON subregion of reversal cells actively responds to luminance increments, whereas the OFF subregion is unactivated because of the abundant low luminance stimuli (i.e., background), and vice versa under a high luminance distribution. It is possible that LGN cells possess the reversal response, and V1 cells carry forward the reversal response from LGN cells. Moreover, the columnar domains of ON and OFF responses are revealed in layer 4 of cat V1 (Jin et al. 2011; Wang et al. 2015) and the polarity‐specific domains that respond to luminance increments or decrements in the full‐field are revealed in the superficial layers of ferret V1 (Smith et al. 2015). The luminance domains have been identified in V2 of cats and monkeys (Tani et al. 2003; Wang et al. 2007). Most of the reversal cells we investigated may be recorded from V1 luminance domains. The reversal cells that have negative or positive ON–OFF indices may be located in dark‐ or bright‐preferring domains in layers 4 and 2/3. The bimodal distribution of the ON–OFF indices of reversal cells (left panel of Fig. 9C) suggests this possibility. If so, the concept that these cells in layers 4 and 2/3 have the reversal response pattern to the change in luminance distribution is understandable. This explains why complex cells in layers 2/3 exhibit reversal responses.

The V1 contains a small number of cells that respond maximally to intermediate luminances (Peng and van Essen 2005; Geisler et al. 2007; Dai and Wang 2012). The responses of mean cells under a uniform distribution of stimulus luminance (control panel of Fig. 2B) are very similar to these cells. Both kinds of cells are small in number. They are most likely the same group of cells. If so, the current findings demonstrate that their response preferences for intermediate luminances may be systematically modulated by the change in luminance distribution (Fig. 2B). Thus, this modulation indicates that the peak luminance that evokes their maximal responses gradually varies with the luminance distribution or mean luminance (Fig. 2B, E). The mechanism responsible for generating the modulation should occur mainly in the cortex because the preference for intermediate luminances is thought to emerge in V1 (Peng and van Essen 2005).

Moreover, a portion of V1 cells has a substantial disinhibitive or counter‐suppressive region far from their classical RF (Li and Li 1994; Sillito et al. 1995; Walker et al. 2002; Wang et al. 2009). The facilitative effects of this counter‐suppressive region are thought to enable the cells to extract additional visual information, such as the average luminance and gradient, from the far surround region (Li et al. 1991; Li and Li 1994; Wang et al. 2009). The luminance changes of the large stimuli we used probably activated the counter‐suppressive region of the V1 cells after they rapidly adapted to the constant contrast carried by these stimuli (Hu et al. 2011). The coactivation of RF surround disinhibitory and center excitatory mechanisms has been shown to enhance V1 response and generate emergent response to originally ineffective orientations in both simple and complex cells. The synchronized activities of cell pairs having cross‐oriented RFs uncover the functional connections underlying the processes (Sillito et al. 1995). These surround disinhibitory mechanisms and the interplay with center excitatory mechanisms may subserve the responses of reversal cells to luminance distributions.

Another potential mechanism is the adaptation that frequently occurs in the sensory systems. In the visual system, adaptation increases responses of visual cells when the input signals are weak to enhance the ratio of response signal to noise, and decreases the responses when the input signals are strong to avoid the saturation of responses (Baccus and Meister 2004; Kohn 2007; Wark et al. 2007; Rieke and Rudd 2009; Carandini and Heeger 2012). The adaptive effects of neuronal responses to the stimulus luminance intensity can be observed in the example responses (LRFs) of the neurons shown in Figures 2A, C, 5A, B, 7B, D, 8A, B. The maximal firing rates of these neurons during the presentation of a low luminance distribution (weak input signals) were higher than those obtained during a high luminance distribution (strong input signals). This is also observed in the population data of the reversal cells (Fig. 5E). The linear increase of luminance gain (*L*_50_) with the increase in the mean luminance of stimulus distribution in both reversal and shift cells (Fig. 5C, D) suggests the adaptive responses to luminance distribution. These data illustrate that V1 neurons adjust their responses to luminance changes by adapting to the luminance distribution when the global distribution changes. The rightward shift in the LRF of V1 shift cells to different luminance distributions is similar to the adaptive responses of the V1 cells to contrast (Ohzawa et al. 1982; Bonds 1991; Hu et al. 2011), indicating that adaptation occurs in the responses of V1 shift cells to luminance distributions. Furthermore, the adaptation to the statistics of luminance distribution probably contributes to these dynamic adjustments of responses because the asymmetry of a luminance distribution is crucial to the adaptive responsiveness (Fig. 7). The responses of reversal cells to luminance decrements may be desensitized or inactivated when they adapt to a HDS that are distributed in a low luminance range because the corresponding luminance distribution contains numerous dark stimuli with luminances close to the mean background luminance. The highly dense dark stimuli inhibit responses to luminance decrements; however, they do not inhibit responses to luminance increments. In this case, only the machinery that detects luminance increments actively works. Analogously, in the case of a HDS distributed in a high luminance range, the responses of reversal cells to luminance increments are inhibited because the luminance distribution contains a substantial amount of bright stimuli with luminances close to the mean background luminance, thus they only respond to luminance decrements. This is supported by the fact that reversal cells respond to both luminance decrements and increments when the mean luminance of a distribution is in a certain intermediate range (Fig. 3). Because neither dark stimuli nor bright stimuli are absolutely dominant in the intermediate luminance distribution, responses to luminance decrements and increments are not inhibited. The adaptation of responses to different luminance distributions must also be involved in mean cells. The adaptive responses to luminance changes have been demonstrated in LGN cells (Mante et al. 2005) and may be further enhanced in V1.

In view of the previously described scenarios, the neural processes that occurred within V1 must play roles in producing the neuronal response behaviors identified in the current study. The long‐range horizontal connections in V1 may contribute to the processes because the horizontal connections in V1 (Gilbert and Wiesel 1983, 1989; Bringuier et al. 1999; Smith and Kohn 2008) have been thought to participate in the generation of responses to luminance changes (Komatsu et al. 2002; Kinoshita and Komatsu 2001; Roe et al. 2005; Hung et al. 2007; Huang and Paradiso 2008). However, the intracortical mechanism is not the sole mechanism that contributes to V1 responses to luminance changes, and subcortical inputs have been suggested to be involved in the process (Dai and Wang 2012; Li and Wang 2013; Wang et al. 2015). The responses of a V1 cell to luminance changes that occurred in a large region should be built up from the inputted responses of the LGN and retina cells to the luminance changes that occurred in the small regions. Therefore, both subcortical inputs and intracortical mechanisms must be involved in the process.

It is interesting to note that the addition of several extra stimuli with high luminance to the set of stimuli with a uniform distribution of luminance (control) caused the dramatic effect of the LRF reversal from the increasing profile (control panel of Fig. 5A) to the decreasing profile in the reversal cells (H1 panel of Fig. 5A). The increase in the number of stimuli brighter than the mean luminance causes more stimuli to be distributed in the high luminance range and the mean luminance to change to a high level. In this case, a reversal cell becomes less sensitive to luminance increments but more sensitive to luminance decrements under a high luminance distribution by the adaptation mechanism previously discussed. Moreover, as Figure 3 shows, if a HDS was investigated along a luminance dimension in a step smaller than that used in the current study, a reversal cell would be found to respond equally to both luminance decrements and increments when the HDS distributed in a specific intermediate range. This intermediate range is different for different reversal cells (Fig. 3). Increasing several more stimuli with low or high luminances will break the balance in which a reversal cell responds to both luminance increments and decrements in the intermediate range of luminance distribution (e.g., Fig. 3). This asymmetry of the resultant luminance distribution causes this cell to exhibit more preferring luminance increments or decrements under the distribution where low or high luminance stimuli are more dominant.

### Functional significance of reversal cells, mean cells, and shift cells

The findings that there are unusual and dynamic changes in the response preferences of reversal cells and mean cells for luminance changes under different luminance distributions indicate the remarkably efficient coding strategy of V1 neurons. These dynamic responses may be established in several presentations by a set of stimuli (Fig. 8G). The activities of a population of cells (Gutnisky and Dragoi 2008; Wang et al. 2011; Benucci et al. 2013) should support the perceptual adaptation of subjects to sudden changes in ambient luminance. The salience of bright stimuli in a dark environment (or dark stimuli in a bright environment) represented by reversal cells and the prevailing mean luminance represented by mean cells are important because they enable subjects to sense the salient targets as the global light distribution abruptly changes. These functional roles may facilitate the perception of subjects to visual stimuli. These neurons signal bright targets in a dim environment and dark targets in a light environment. Shift cells may play a role in precisely discriminating the most rapidly changing luminance by shifting their dynamic responses that are the most sensitive to luminance changes with the change of global luminance distribution. Functional roles of reversal cells and mean cells are more significant than shift cells in detecting a substantial magnitude of light change when a drastic variation in light intensity occurs in the environment.

## Conflict of Interest

The authors declare no conflict of interest.
